# Anterior chest hump in adolescent idiopathic scoliosis- questionnaire evaluation

**DOI:** 10.1186/1748-7161-7-S1-O11

**Published:** 2012-01-27

**Authors:** K Watanabe, N Hosogane, K Chiba, Y Toyama, M Matsumoto

**Affiliations:** 1 Keio University, Tokyo, Japan

## Background

Thoracic hump, shoulder imbalance, lumbar hump were known as trunk deformities associated with scoliosis bothering patients. In addition to these deformities, anterior chest hump (ACH) occurring at the distal end of the anterior chest wall on the convex side, may be another noteworthy problem. We evaluated how much patients with adolescent idiopathic scoliosis (AIS) were bothered by ACH based on our original questionnaires conducted in patients undergoing surgery.

## Materials and methods

Fifty-seven AIS patients (all females) who underwent surgical treatment were included in this study. A mean age at the time of surgery was 17.2±4.6years. A mean preoperative Cobb angle of the main thoracic curve was 58±14°. The questionnaire consisted of five numerically-rated questions asking how much the patient was bothered by thoracic hump, lumbar hump, ACH, waist asymmetry, and shoulder imbalance. The perception about the deformities was scored from 0 (None) to 10 (Worst). Correlation between the score of ACH and thoracic Cobb angle was also evaluated.

## Results

The mean score of ACH was 3.8±3.4points, which was almost equal to that of lumbar hump (3.9±3.7points). The mean scores were 5.8±3.9points in waist asymmetry, 5.7±3.5points in thoracic hump, and 4.9±3.4points in shoulder imbalance. The score of ACH was significantly correlated with thoracic Cobb angle (correlation coefficient: 0.43, p<0.001).

## Conclusions

The results indicated that ACH was bothering problem for AIS patients similarly to other trunk deformities. Since the perception of trunk deformity reported to be strongly associated with patient satisfaction for treatments [1], ACH should be noted as one of the trunk deformities bothering patients.
